# English Criticism

**Published:** 1892-05

**Authors:** 


### Editorial.

### ENGLISH CRITICISM.

THE quotations made under the heading of "Selections," from the March number of the *British Journal of Dental Science*, will doubtless be read with interest by those not familiar with English criticisms. It is usually a very excellent rule to permit letter-writers in journals to express their feelings on any given subject and maintain a dignified silence as the most fitting reply. The present instance seems to require attention, not so much from the intrinsic value of the communications as their representative character.

They are but another evidence of the jealous feeling, not yet obliterated from the professional mind in any country, and which must be regarded by men of broad views as a serious detriment to progress.

The criticisms made by Dr. W. Mitchell, of London, and published in the January issue of this journal, it is unnecessary to quote here *in extenso*, but we give the paragraph which seems to have aroused the ire of the dental fraternity in England.

"American dental colleges, like almost all other earthly institutions, are open to improvement, and I have no doubt but their progressive superiority will be demonstrated in the future, as it has undoubtedly been in the past, continuing to furnish the pattern that is so poorly copied on this side, and which, I am sorry to say, at present is more of a cheap burlesque than a worthy replica; and while ridicule and poverty-stricken wit has taken the place of argument and logic in the European press, the fact remains that more bogus diplomas are held and more questionable means resorted to to obtain legitimate degrees by men on this side of the Atlantic than upon the other, and had it not been for the European market these diploma-mills would have died a natural death."

While these expressions are severe, they do not seem to warrant the bitter onslaught made in the responses. The deans of all the colleges of England seem to have arisen as one man to repel the charge that American colleges are in most respects better than those they represent. With this we have nothing to do, neither have we personal knowledge sufficient to formulate an opinion. Comparison between schools of the same country, as well as those of widely different nationalities, must always be liable to error, from the fact that, unless there is an intimate acquaintance with details, not possible to an outsider, the conclusion arrived at will be imperfect.

Had the criticism quoted been made by an American writer, it is safe to presume no notice would have been taken of it, for, as "S." says in his letter, "Mr. W. Mitchell . . . is a member of the British Dental Association and of the Odontological Society of Great Britain. It is *this fact alone*, of course, *which lends the least importance to Mr. Mitchell's writings*." (Italics ours.) The inference of this is obvious, that Mr. Mitchell has given expression to knowledge obtained through intimate relations with the best elements of English dentistry.

It cannot be regarded of vital importance whether English schools are better or worse than American. They serve the purpose of the profession there, and no doubt accomplish it to the satisfaction of all concerned. The American colleges, on the other hand, have equally endeavored to build up a system of their own, adapted to the peculiar environments of their own country. The development of this system has been accomplished under very trying circumstances,—prejudice at home, criticism from abroad, and fraudulent dealings in worthless diplomas, for which neither the colleges of the United States or the government were responsible. A sample of this kind of criticism we reproduce here from the journal alluded to: "When all *our questionable means* to secure a diploma of one of *our own colleges*, which, after all, we are told, is only, well, 'a cheap burlesque,' has failed, Brother Jonathan receives us with open arms, and for a few dollars grants us a genuine bogus article."

This unfounded statement is but a repetition of much that has been published on the Continent; and when Dr. Mitchell says "that more bogus diplomas are held and more questionable means resorted to to obtain legitimate degrees" by men in Europe than in this country, he simply states an absolute truth.

The experience of the writer through the most serious period of the "bogus-diploma business" led to the conviction that this nefarious trade was conducted in this country through agents in Europe, and that it was for a time impossible to procure a specimen of these fraudulent issues on this side of the Atlantic to be used as evidence. In an active personal search in Philadelphia at one period for legal proof, not a single diploma could be secured, and but one person found who held one. The then mayor of Philadelphia informed the writer that he had made every effort, through his police force, to secure one, and failed. At this same period Europe was being flooded with them. It is needless to say—as it is a matter of history—that the evidence was eventually secured, and the orginal, too, of this infamous business was imprisoned, and finally died. His bad work has been taken up by others, and from some mysterious source these fraudulent productions are still sent to Europe, or, what is more likely to be the case, manufactured there.

It is also one of the experiences that letters are constantly being received, as they have been for a score of years, asking for diplomas *in absentia*, and while these applications have not been confined to any particular country in Europe, they have come in greater proportion from Germany. During the same period there have been but few received from citizens of the United States, and none at all for the past six years. It is absolutely impossible now, with our present stringent laws, for one to practise under such a document, and we believe this to be equally true of England.

The assertion is made by one of the correspondents (Kosmos) that "the engrossing topic in dental circles of repute is how to raise the quality of the teaching in American schools so as to assimilate it to the British standard." This is evidently based on defective information. The National Association of Dental Faculties represents the entire teaching-force in the United States and Canada, and from this body all proposed changes must originate. No such proposition has ever been before it, nor has the idea ever been suggested that schools under their jurisdiction should be made to conform to the English standard. The question has been raised as to the propriety of increasing the requirements for entrance,—not in accordance with the English idea, but to a standard consistent with American experience and progress. In this matter each country must be governed by its peculiar conditions, and in this direction criticism has no place. Indeed, the feeling of many well-educated men in dental and medical circles is that a very high standard for preliminary examinations is a mistake. This opinion has repeatedly been confirmed in dental teaching, and it has been demonstrated, over and over again, that the man educated in all that the higher literary colleges can bestow has no special advantage over the one of a common-school education; and oftentimes is found lacking in those qualities that go to make the practical professional man.

That which seems to trouble the writer quoted is the fact that

Dr. Mitchell states that "the best and most progressive students, after completing their course on this side (Europe), . . . seek the halls of learning in America." Exactly why this should trouble our brethren in England is a problem, unless it be ascribed to an envious feeling of the work here. We are not aware that it is a common practice for persons to leave Great Britain for this country to secure a dental education; but we have had personal acquaintance with many who have been on this side to take *post-graduate courses* in various institutions. It seems that this should indicate a superior degree of intelligence, a desire to broaden the knowledge already obtained by interchanging experiences with others; and by no course of reasoning can it be construed into a reflection of the training previously received. These men have invariably been of high character, and whether they have gained much or little here, they have left a most excellent impression on the dental fraternity, and their presence has aided in correcting ideas in regard to dental education abroad.

What is true of England is equally true of Europe generally. These students do not come for the diploma, for that to them is of but little value, but to acquire other modes of practice peculiar, it may be, to this country. For the same reason, large numbers of educated men, literary, scientific, and professional, leave America and seek the schools of Europe. The man who refuses to remain locked up in narrow ideas, whether these be generated in the United States, Great Britain, or Europe, is one who takes the best possible course to secure a larger intelligence and ability to practise any of the callings of interest in the world.

Criticism such as this is, therefore, out of place; but if it must be adopted, we would kindly suggest to our English confrères that vituperation does not constitute an elevated standard of English writing from the stand-point of American intelligence, nor is it ever likely to command the thoughtful respect of their co-laborers in other lands.

