# Correction

**Published:** 1892-06

**Authors:** 


### CORRECTION.

In the editorial headed "English Criticism," in the May number, the name of the *British Journal of Dental Science* was substituted for *The Journal of the British Dental Association.* It is presumed the intelligent reader observed the error and made the proper correction.

